# Can We Use Home Sleep Testing for the Evaluation of Sleep Apnea in Obese Pregnant Women?

**DOI:** 10.1155/2019/3827579

**Published:** 2019-08-04

**Authors:** Francesca L. Facco, Victoria Lopata, Jennifer M. Wolsk, Sanjay Patel, Stephen R. Wisniewski

**Affiliations:** ^1^Department of Obstetrics, Gynecology, and Reproductive Sciences, University of Pittsburgh School of Medicine, Pittsburgh, PA, USA; ^2^University of Pittsburgh, Pittsburgh, PA, USA; ^3^Center for Sleep and Cardiovascular Outcomes Research, University of Pittsburgh School of Medicine, Pittsburgh, PA, USA

## Abstract

**Objective:**

To evaluate the performance of a type III home sleep testing (HST) monitor including its autoscoring algorithm, in a population of obese pregnant women.

**Methods:**

This was an ancillary study of an ongoing prospective study of obese (BMI of ≥30) pregnant women. For the primary study, women undergo serial in-lab polysomnograms (PSG) during pregnancy. Sleep apnea was defined as an apnea hypopnea index (AHI) of ≥ 5 events/hour. A subgroup of women were asked to wear an ApneaLink HST device for 1 night, within 2 weeks of a late pregnancy PSG (≥ 28 weeks' gestation). The AHI obtained from PSG was compared to the AHI from the HST via autoscoring (HST-auto) as well as the AHI via technician scoring (HST-tech). We calculated Shrout Fleiss Fixed correlation coefficients (ICC) and looked at positive-positive and negative-negative agreement.

**Results:**

43 women were recruited and we obtained 30 valid HST. The mean PSH AHI was 3.3 (±3.2, range 0.5-16.6). Six (20%) women had a positive PSG study. ICCs were 0.78 for HST-auto versus HST-tech, 0.76 for HST-auto versus PSG, and 0.70 for HST-tech versus PSG. Categorical agreement was also strong, with 24/30 (80.0%) for HST-auto versus HST-tech, 25/30 (83.3%) for HST-auto versus PSG, and 23/30 (76.7%) for HST-tech versus PSG.

**Conclusion:**

In obese women evaluated in late pregnancy, we found relatively high intraclass correlation and categorical agreement among HST-auto scores, HST-tech scores, and in-lab PSG results obtained within a two-week window. These results suggest that HST may be used to screen pregnant women for OSA.

## 1. Introduction

Obstructive sleep apnea (OSA) in pregnancy has been associated with adverse maternal and neonatal outcomes [1–3]. In a large epidemiologic study of OSA in pregnancy, about 15% of women with a BMI ≥ 30 had evidence of sleep apnea in the first trimester pregnancy, and the rate doubles to 30% when retested in mid-pregnancy [1]. However, there are limited data on best practices to screen for and treat OSA in pregnancy. Type III home sleep testing (HST) devices with autoscoring capabilities may lessen the burden of testing for OSA in pregnancy; however data regarding their reliability in pregnancy are limited. If ongoing research results continue to support the role of screening for sleep apnea in pregnancy, it will be important to optimize the use of HST to help care providers quickly triage which pregnant women are at greatest need of possible treatment of referral to a sleep expert as long delays for in-lab PSG cannot be tolerated with a time-limited situation as pregnancy.

The American Academy of Sleep Medicine comments that although it is less sensitive than polysomnography in the detection of OSA, a type III HST can be ordered by a physician for the diagnosis of OSA when the physician has determined that the patient does not have other medical conditions or risk for other sleep disorders that would preclude the use of an HST and has identified signs and symptoms that indicate an increased risk of moderate to severe OSA, rather than mild OSA [4]. Epidemiologic data demonstrate that the vast majority of OSA identified in pregnancy is mild in severity; however not extending the option of home sleep testing to pregnant women could significantly limit the ability to diagnose and treat OSA in this vulnerable patient population [1]. Therefore, it is imperative that we do more to understand how HST performs in pregnancy. Currently there are little data on the use of HST in pregnancy, specifically on how autoscoring algorithms perform in pregnancy compared to both technician review of HST recordings and in-house polysomnography.

The ApneaLink (ResMed, Sydney, Australia) is a pocket-sized type III home sleep testing device. It consists of 3 recommended and validated sensors for measuring respiration: a nasal pressure transducer (that measures nasal airflow and waveform, which are needed for the detection of apneas and hypopneas and airflow limitation), a thoracic inductance plethysmography band that measures respiratory effort for distinguishing central from obstructive apneas (and as a back-up for the nasal pressure signal), and finger pulse oximetry (to quantify the level and duration of oxygen desaturation). The recorded signals can be analyzed automatically by the ApneaLink software platform to generate an “autoscore” but can also be reviewed, edited, and rescored by a sleep technician within the same software package.

The objective of this study was to evaluate the performance of the ApneaLink HST, including the autoscoring algorithm, to diagnose OSA in a population of obese pregnant women.

## 2. Methods

This was an ancillary study of an ongoing prospective study of OSA in obese pregnant women with a singleton pregnancy. Women with a known preexisting diagnosis of sleep apnea were excluded from this study. For the primary study, women with a BMI ≥30 kg/m^2^ are recruited during pregnancy and undergo an in-lab polysomnogram (PSG) before 21 weeks' gestation (early pregnancy) and then again at 28-32 weeks' gestation (late pregnancy). OSA is defined as an apnea hypopnea index (AHI) of ≥ 5 events/hour. A subgroup of these women was then asked to wear an ApneaLink home sleep testing device for 1 night, within 2 weeks of the late pregnancy PSG study. Written informed consent was obtained from all individual participants included in the study.

### 2.1. PSG Scoring

All in-lab PSGs were performed at a research lab using Harmonie Version 6.2e software. Respiratory events were scored on PSG with apneas defined as ≥ 90% reduction in airflow for a minimum of 10 seconds and hypopneas defined as ≥ 30% reduction in airflow for a minimum of 10 seconds, associated with ≥3% reduction in oxyhemoglobin saturation.

All sleep technologists (n=3) who scored PSG data for this study complete monthly scoring reliabilities as part of the American Academy of Sleep Medicine Interscorer Reliability program and have an average percent agreement of 94.4% (http://isr.aasm.org).

### 2.2. ApneaLink Scoring

ApneaLink recordings were considered valid if they had ≥ 4 hours of adequate nasal flow and oximetry signals. We used the ApneaLink autoscoring algorithm to obtain an autoscore AHI (HST-auto). We utilized the software's AASM 2012 autoscore algorithm which employs apnea and hypopnea definitions consistent with the PSG definitions noted above.

After an autoscore was obtained, all ApneaLink recordings were then reviewed, edited, and rescored, in a blinded fashion, by the same team of sleep technologists who had scored the original PSG recording. The AHI calculated by this review was labeled as HST-tech.

### 2.3. Statistical Methods

To compare performance of HST-auto versus HST-tech, HST-auto versus PSG, and HST-tech versus PSG, we produced scatterplots, calculated a Shrout Fleiss Fixed intraclass correlation coefficient (ICC), and produced Bland-Altman plots (designed to compare two measurement techniques) for each pair. In addition, we converted scores into categorical data (AHI≥ 5 = positive; <5 = negative) and then looked at positive-positive and negative-negative agreement. We opted not to use the Kappa statistic, as originally planned, due to the small N and 0 cell values.

## 3. Results

A total of 43 women were recruited to participate in the HST study. Their HSTs were excluded if they had less than 4 hours (240 minutes) of nasal flow or oximetry signal (N=13) and/or were not returned within two weeks of the late pregnancy PSG (N=1). Demographics of the 30 women with a successful study is shown in Table 1.

**Figure 1:**
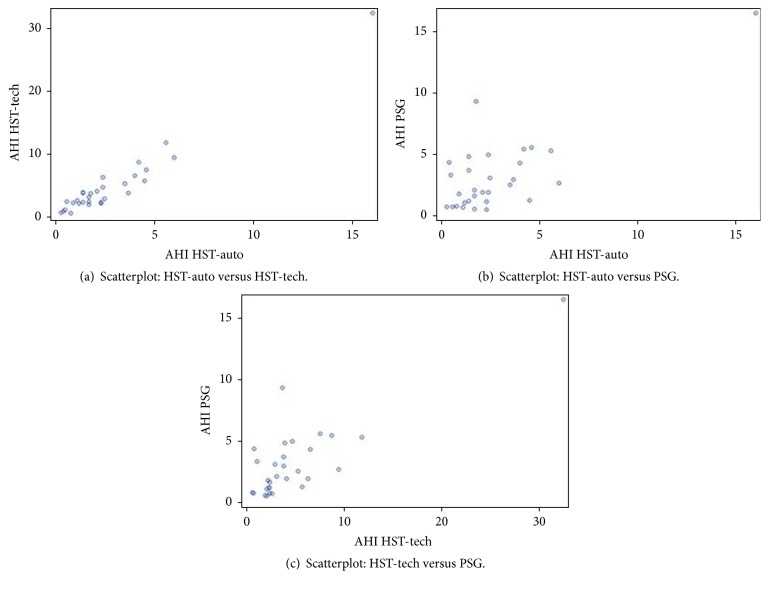
(a)–(c) Scatterplots of AHI comparisons. (a) HST-auto vs. HST-tech, (b) HST-auto vs. PSG, and (c) HST-tech vs. PSG.

The mean AHI for PSGs was 3.3 (±3.2, range 0.5-16.6). Of the 30 women, 6 (20%) had a PSG study consistent with mild or moderate sleep apnea. The mean time difference between PSG and HST was 2.4 ± 3.1 days. There was good agreement between each pair of tests (Figures 1(a)–1(c)). Intraclass correlation coefficients (ICCs) were 0.78 for HST-auto versus HST-tech, 0.76 for HST-autoscore versus PSG, and 0.70 for HST-tech versus PSG.

Categorical agreement was also strong, with 24/30 (80.0%) for HST-auto versus HST-tech, 25/30 (83.3%) for HST-auto versus PSG, and 23/30 (76.7%) for HST-tech versus PSG (Tables 2(a)–2(c)). Of the 24 women with a negative PSG (AHI < 5), 23 (95.8%) were also negative by HST-auto, while 19 (79.2%) were also negative by HST-tech. The discordant negative studies are listed in Table 3.

Data from all the positive PSG are presented in Table 4. Of the 6 positive PSGs, 2 were classified as positive by HST-auto while 4/6 were identified as positive by HST-tech. Two studies were discordant by both HST-auto and HST-tech. 50% of cases of mild OSA misclassified by HST scoring (auto or tech) had HST AHI values of > 4 but < 5 (Table 4).

**Figure 2:**
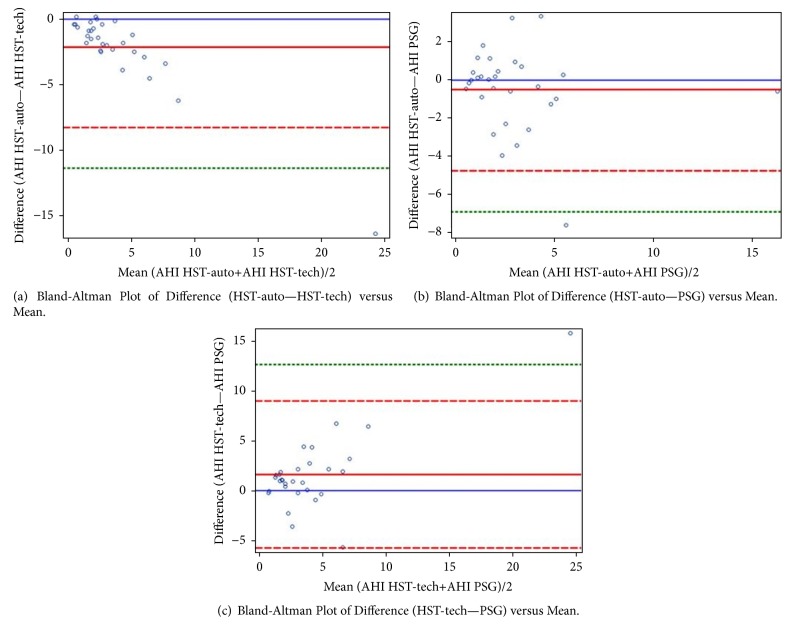
(a)–(c) Bland-Altman plots of AHI comparisons. (a) HST-auto vs. HST-tech, (b) HST-auto vs. PSG, and (c) HST-tech vs. PSG. Blue solid lines are a zero reference, red solid lines are the mean difference, dashed red lines represent the ±2 standard deviations from the mean, and dashed green line represents the ±3 standard deviations from the mean (for image resolution, given ranges, not all standard deviation lines were graphically presented).

Bland-Altman plots (Figures 2(a)–2(c)) provide additional information for comparing each pair of tests. As shown, HST-tech scores were consistently higher than HST-auto scores, with the difference increasing as the mean increased. HST-auto and PSGs, in contrast, tracked relatively well, with the distribution centered around 0. HST-tech did not track quite as well as HST-auto relative to PSG.

## 4. Conclusion

In this study of obese women in late pregnancy we found relatively high intraclass correlation and categorical agreement among HST-auto scores, HST-tech scores, and in-lab PSG results obtained within a two-week window. Notably in our study autoscoring had more cases of underdiagnosis of OSA, while tech scoring yielded more cases of overdiagnosis.

## Figures and Tables

**Table 1:** Participant demographics.

Maternal age	27.3 ± 4.2

BMI (kg/m^2^)	39.5 ±6.0

Gestational age at late pregnancy sleep study, in weeks	29.2 ± 1.0

Self-report of frequent snoring	7/30 (23.3%)

Race	
African-American	22/30 (73.3%)
Caucasian	6/30 (20.0%)
Multiracial	2/30 (6.7%)

**Table:** (a) Categorical agreement: HST-auto vs. HST-tech with categorical agreement in italic.

	HST-tech		
HST-auto	AHI <5	AHI≥5	

AHI <5	*21*	6	27

AHI≥5	0	*3*	3

	21	9	

**Table:** (b) Categorical agreement: HST-auto vs. PSG with categorical agreement in italic.

	PSG		
HST-auto	AHI <5	AHI≥5	

AHI <5	*23*	4	27

AHI≥5	1	*2*	3

	24	6	

**Table:** (c) Categorical agreement: HST-auto vs. PSG with categorical agreement in italic.

	PSG		
HST-tech	AHI <5	AHI≥5	

AHI <5	*19*	2	21

AHI≥5	5	*4*	9

	24	6	

**Table 3:** Data from negative PSG (AHI< 5) that were discordant on HST (n=5/24 total negative).

HST-auto	HST-tech	PSG	AHI-auto to PSG	AHI-tech to PSG
4.5	5.7	1.3	Concordant	Discordant

3.5	5.3	1.6	Concordant	Discordant

2.4	6.3	1.9	Concordant	Discordant

4.0	6.5	4.3	Concordant	Discordant

6.0	9.4	2.7	Discordant	Discordant

**Table 4:** Data from all positive (AHI ≥5) PSGs (n=6 total positive PSG).

HST-auto	HST-tech	PSG	AHI-auto to PSG	AHI-tech to PSG
5.6	11.8	5.3	Concordant	Concordant

16.0	32.4	16.6	Concordant	Concordant

4.2	8.7	5.5	Discordant	Concordant

4.6	7.5	5.6	Discordant	Concordant

2.4	4.7	5.0	Discordant	Discordant

1.8	3.7	9.4	Discordant	Discordant

## Data Availability

The data from this research is not publically available at this time but will abide by all NIH regulations about data availability as this was a NIH funded study.

## Additional Points

*Study Limitations.* The main limitation of our study is that while we had an adequate sample size to assess correlation of raw AHI values, given the overall prevalence of sleep apnea in this study (20%), our precision for estimating the positive or negative predictive values of HST scoring at any specific cutoff values (e.g., ≥5 or ≥4) is limited. Additionally, since we allowed an up to 2-week interval between PSG and HST (though mean was only 2.4 days), some variability seen between the PSG and HST values may be attributed to changes in respiratory events due to advancing gestation and night to night variability, not the differences in the sleep assessment modalities themselves.

The validity of the ApneaLink HST for diagnosis of OSA in a nonpregnant population has been demonstrated by multiple investigators. One of the largest validation studies recruited 149 individuals referred to a sleep center due to habitual snoring or witness apneas. They wore the ApneaLink concurrent with their in-lab PSG and also repeated the ApneaLink at home within one month [5]. The mean AHI by PSG in this study was 25.7 ± 22.1, with over 85% of participants having an AHI of ≥5, about a third of whom had mild disease (AHI 5-14.9). In this population, the investigators reported excellent intraclass correlations between the ApneaLink with manual scoring or with AASM 2012 autoscoring and the PSG, both when worn in lab and at home. While the manual scoring was most accurate the sensitivity and specificity of manual versus autoscoring were comparable (sensitivity and specificity of Apnea Link worn at home: manual scoring 93.0 % and 61.9%, resp.; autoscoring 85.9 % and 66.7%, resp.). In another study of a community based cohort of 100 individuals (ages 21-80) investigators compared automated versus manual scoring of the ApneaLink device [6]. In this study 6% of participants had no sleep apnea and 14% had mild OSA. The investigators found an excellent correlation (coefficient 0.968; 95% CI, 0.952-0.978). Furthermore, examining manual vs. automated results by OSA severity (normal, mild, moderate, or severe) they found an overall misclassification rate of 22%, with < 10% representing a clinically significant misclassification likely to have an impact on therapeutic decisions.

Two prior studies have looked at HST performance specifically in pregnancy. O'Brien et al. looked at the Watch-PAT 200 device in a group of 31 women ≥28 weeks' gestation. Women in this study wore the Watch-PAT the same night as an in-home PSG (Braedon, Ontario, Canada) [7]. Watch-PAT proprietary software was used for automated scoring. The software applies an algorithm to analyze a peripheral arterial tonometry signal amplitude along with the pulse and oxyhemoglobin saturation to estimate the AHI. The mean PSG AHI was 5.4+/- 8.5, and the prevalence of OSA in this study was similar to ours at 26%. They found an excellent correlation between Watch-PAT and PSG AHI and reported a sensitivity of 88% and a specificity of 86% for sleep apnea at a Watch-PAT AHI threshold of 5.

Sharkey et al. examined the Apnea Risk Evaluation System (ARES) worn concurrently with in-laboratory PSG in a group of 16 pregnant women who were referred to a sleep center for clinical suspicion of OSA [8]. Raw ARES Unicorder data were edited by a physician reader to determine sleep onset and offset times and to remove obvious artifacts such as device removal and excessive head movements and dropped SpO2 signals. Additionally, edited data files were processed using the ARES Insight software (Watermark Medical), which applies automated algorithms to raw data to identify arousals associated with changes in oxyhemoglobin saturation, pulse, head movement, effort of breathing and airflow, and snoring levels during sleep. The average gestational age of participants was 28.6 ± 6.3 weeks (range 17 to 38 weeks). The median AHI was 3.1 events/h of sleep (interquartile range [IQR] = 0.4 to 8.0), and 6/16 women had an AHI ≥5 (37.5%). They also observed a high correlation between ARES AHI and PSG AHI. The ARES algorithm identified all 6 women diagnosed with OSA by PSG but also resulted in ARES AHI values ≥ 5 in 5 out of 10 women who did not have PSG AHI values ≥ 5 events/h.

One difference between our study and these two prior studies of HST in pregnancy is that we did not do concurrent recording with the ApneaLink, but rather we tested the ApneaLink in a real-world scenario having the participant take the device home and self-administer it. Nevertheless, the results we reported using the ApneaLink in these real-world conditions are consistent with the results of these prior reports of HST in pregnancy. Additionally, our study examined both the output of autoscoring and the technician scoring (blinded) of the ApneaLink device.

Understanding the validity of home sleep testing in pregnancy is of critical importance given that the special circumstances of pregnancy (e.g., time limited, other children in the home) often make the burdens of in-laboratory PSG unfeasible. Overall our data as well as data from prior reports suggest that home sleep tests in pregnancy can be a useful tool for the initial evaluation of OSA in pregnancy, even though the majority of OSA cases in pregnancy are mild in severity. How best to deploy home sleep testing in pregnancy and how to incorporate autoscoring versus manual scoring algorithms into clinical practice are questions that warrant further investigation.
